# Enrichment of Germline Cystic Fibrosis Transmembrane Conductance Regulator (CFTR) Pathogenic Variants in Patients With Solid Tumors: Evidence for Increased Cancer Risk

**DOI:** 10.1002/ppul.71360

**Published:** 2025-10-28

**Authors:** Tyler Shugg, Katherine A. Gallaway, Nick Powell, Todd C. Skaar, Steven M. Bray, Leigh Anne Stout, Abi Colwell, Cynthia D. Brown, James E. Slaven, Emma M. Tillman

**Affiliations:** ^1^ Division of Clinical Pharmacology, Indiana University School of Medicine Indianapolis Indiana USA; ^2^ Fountain Life Indianapolis Indiana USA; ^3^ Division of Medical and Molecular Genetics, Indiana University School of Medicine Indianapolis Indiana USA; ^4^ Indiana University Health Precision Genomics Indianapolis Indiana USA; ^5^ Division of Pulmonary, Critical Care, Sleep, and Occupational Medicine Indiana University School of Medicine Indianapolis Indiana USA; ^6^ Department of Biostatistics and Health Data Science Indiana University School of Medicine Indianapolis Indiana USA

**Keywords:** cancer, CFTR, cystic fibrosis

## Abstract

**Introduction:**

Wide‐spread use of cystic fibrosis (CF) transmembrane conductance regulator (CFTR) modulators has resulted in a dramatic increase in life expectancy of people with CF. Historically, *CFTR* carrier status has been thought to be benign; however, data are emerging that dysfunction of *CFTR* may play a role in cancer risk. The aims of this study were to (1) determine the frequency of germline pathogenic *CFTR* gene variations in a cohort of patients with cancer, and (2) investigate associations between germline pathogenic *CFTR* variants and established germline somatic oncogenic driver mutations.

**Methods:**

The Indiana University Adult Precision Genomics (APG) clinical database was queried for all subjects having a germline *CFTR* pathogenic variant (PV). The *CFTR* PVs observed in our cohort were compared to published population minor allele frequencies from the Allele Frequency Aggregator (ALFA) Project then scaled to reflect our APG population.

**Results:**

Data were available for 2141 patients. Based on known minor allele frequencies and the racial distribution of our cohort, we expected 33 subjects (1.5%) to have *CFTR* PVs. However, *CFTR* PVs were present in 71 subjects (3.3%), 2.1‐fold higher than expected (*p* < 0.001). The observed enrichment of *CFTR* PVs varied by cancer type, with *CFTR* PVs being overrepresented in patients with skin and gastrointestinal cancers.

**Conclusions:**

The overall prevalence of *CFTR* PVs in people with solid tumors was higher than expected. These results suggest that carriers of *CFTR* PV may benefit from enhanced cancer screening.

## 1 Introduction

Cystic fibrosis (CF) was once a fatal disease of childhood, but with advances in combination CF transmembrane conductance regulator (CFTR) modulator therapies, children born with CF between 2019 and 2023 have a median predicted life expectancy of 61 years, nearly doubling over the past two decades [1]. Despite remarkable improvements in life expectancy, CF continues to be a chronic multiple organ system disease [2]. As people with CF continue to age, new disease risks associated with adulthood must be considered. Recent data indicate that colorectal cancer incidence is up to 10 times greater and presents earlier in people with CF as compared to the general population [3]. This prompted the CF Foundation to commission a practice guideline for enhanced colorectal cancer screening for people with CF [4]. In addition, recent data indicate that people with CF also have increased relative risk of pancreatic and liver cancers [5, 6, 7], but data are not yet sufficient to recommend increased cancer screening [8, 9, 10, 11].

While historically having a heterozygous *CFTR* pathogenic variant (PV) was once thought to be relatively benign, data are emerging that dysfunction of *CFTR* in either people with CF or carriers of a single *CFTR* PV may increase risk of cancer and other diseases [12, 13, 14, 15]. This is supported by preclinical data showing that the downregulation of CFTR production in animals and in vitro models is associated with increased carcinogenesis [16].

Now that people with CF are living longer, cancer risk is becoming an increasingly important healthcare concern. Thus, the primary aim of this study was to determine the frequency of *CFTR* PVs in a cohort of adult patients with advanced cancer. The secondary aim of this study was to explore biological mechanisms for how *CFTR* PVs may increase cancer risk by investigating associations between germline *CFTR* PVs with (a) known germline cancer predisposition variants and (b) known somatic oncogenic driver mutations.

## 2 Materials and Methods

The Indiana University (IU) Adult Precision Genomics (APG) clinic database contains paired somatic and germline sequencing data from 2422 patients with locally advanced or metastatic solid tumors diagnosed between 2015 and 2023. All people undergoing genomic sequencing within the IU APG clinic are enrolled in a Total Cancer Care IRB protocol that allows future research use of genetic and clinical data. These data, including genomic binary alignment map (BAM) files are returned to the Precision Health Cloud database (Fountain Life, Indianapolis, IN), where they are available for use by clinicians and credentialed researchers. The IU IRB reviewed the specific protocol of this study and deemed it “not human research” as we only accessed deidentified patient data from patients previously consented for the Total Cancer Care protocol. The APG database was queried for all subjects having any *CFTR* PVs, as catalogued in the Clinical and Functional Translation of CFTR (CFTR2) database [17]. The availability of this clinical genomic data defined our cohort as this was a secondary retrospective study using existing data and patients were not prospectively enrolled for the purpose of this evaluation. These CFTR variants were extracted form germline BAM files from clinical WGS (performed as commercially available test from the following laboratories: NantOmics Inc.) (Culver City, CA) [18], Ashion Analytics LLC (Phoenix, AZ) [19], and Caris Life Sciences (Dallas, TX) [20]. using Aldy in a Jupyter Notebook in the Fountain Life Precision Health Cloud environment [21]. Specific CFTR PV coverage is shown in Supporting Information S1: File 1. Only those PV CFTR variants included in CFTR2 were used for classification in our cohort CFTR PV in this study [17].

Patients were excluded for missing clinical data, such as cancer type and diagnosis date, or if somatic and germline sequencing data were unavailable for analysis. In addition to the presence of *CFTR* PVs, we also collected race, sex, age at cancer diagnosis, and cancer type. Specific solid tumor cancer types were categorized into breast, endocrine, gastrointestinal, genitourinary, gynecologic, head/neck, musculoskeletal, neurologic, respiratory/thoracic, skin, or unknown primary. Frequency of *CFTR* PVs were calculated for the total cohort and per specific cancer type. Specific *CFTR* PVs frequencies observed in our cohort were compared to published population minor allele frequencies (MAF) from the Allele Frequency Aggregator (ALFA) Project then scaled to reflect the race and ethnicity of our APG cohort population [22]. For example, the F508del minor allele frequency is 0.00312 in Europeans, 0.00315 in Africans, and 0 in Asians. We used race based MAF to weight to the proportion of our cohort within each race category (96% patients, with 87% being White, 7.3% Black, 1.6% Asian, and < 0.5% American Indian, Alaskan or Hawaiian Native, or multiple races) to estimate the expected frequency of each variant. To explore potential mechanisms for how *CFTR* PVs may increase cancer risk, we reviewed the genomes of *CFTR* carriers for (1) known pathogenic or likely pathogenic germline variants within established cancer predisposition genes as contained in the Ambry CancerNext and Invitae Common Hereditary Cancers panels, and (2) known pathogenic or likely pathogenic somatic cancer driver mutations, as reported in clinical reports from NantOmics, Ashion Analytics, and Caris Life Sciences [18, 19, 20]. The frequencies of somatic cancer driver mutations were compared to those from the Catalogue of Somatic Mutations in Cancer (COSMIC) database, both overall and matched by cancer type.

Statistical analyses were performed to determine if there were significant differences between the CFTR and the non‐CFTR variant groups, using Chi‐Square tests for categorical variables and Student's *t*‐test for age. Analyses were performed using SAS v9.4 (SAS Institute, Cary, NC). Statistical comparisons of *CFTR* PV and somatic cancer driver mutation frequencies between our study cohort and those from the ALFA project and COSMIC database, respectively, were performed using Yate's corrected Chi‐Square tests. The Sidak method was used to correct *p* values for multiple statistical comparisons [23].

## 3 Results

**Figure 1:**
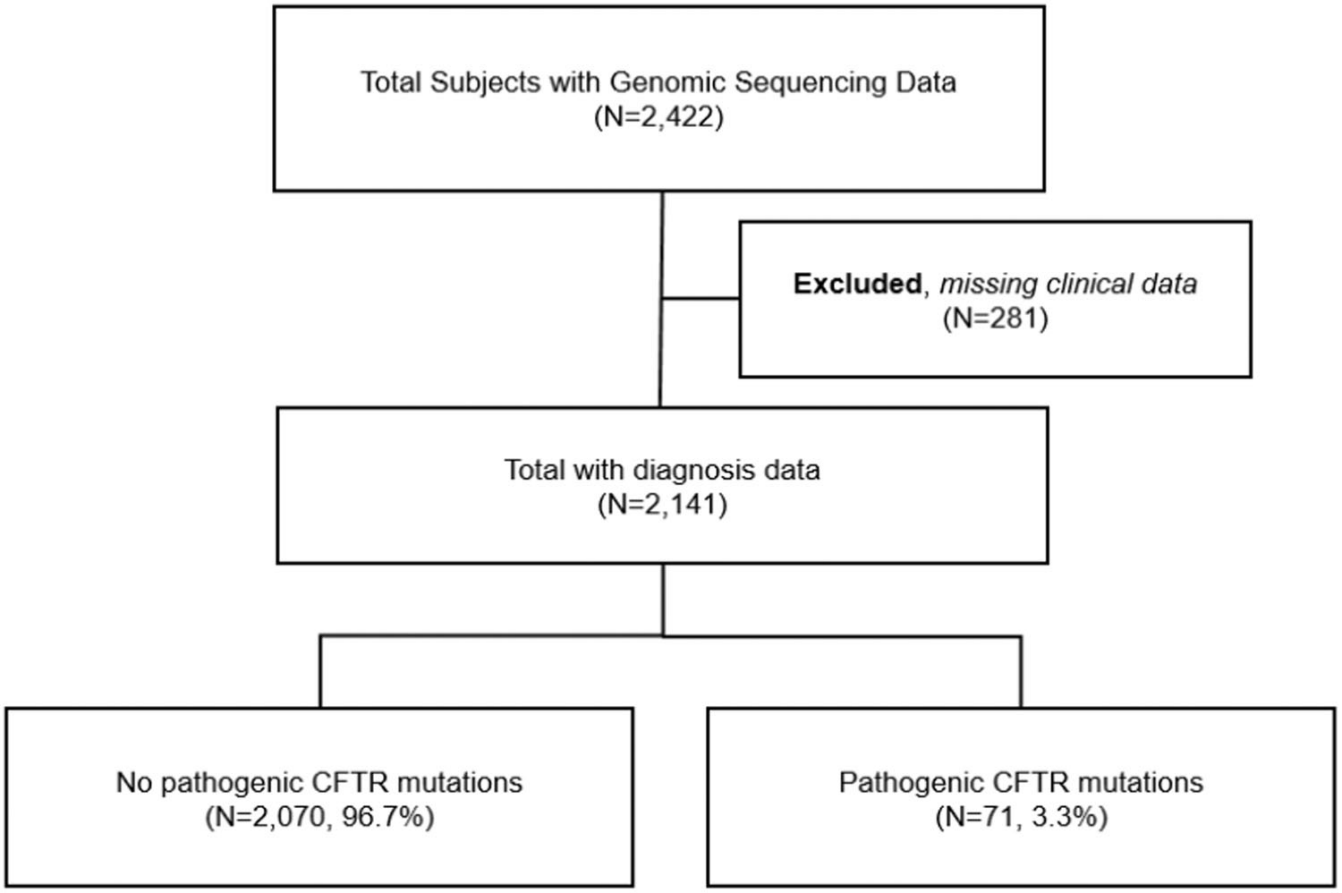
Study Cohort. Study cohort inclusion and exclusion are described. *Based on analysis of standardized residuals, the significant chi‐square p‐value was driven by greater than expected frequencies of *CFTR* PVs in skin (standardized residual: 2.3) and gastrointestinal cancers (2.0) and less than expected in the miscellaneous/undetermined category (−4.0).

The APG cohort had germline and somatic sequencing data for 2422 people with an advanced cancer diagnosis. Clinical data on cancer type and age at diagnosis were missing for 281 people; therefore 2141 people were included in this study (Figure 1). Race of our APG cancer cohort was reported for 96% patients, with 87% being white, 7.3% black, 1.6% Asian, and < 0.5% American Indian, Alaskan or Hawaiian Native, or multiple races. Ethnicity was not reported. Among the 2141 patients included, 51.8% reported male sex, and the average age at first cancer diagnosis was 57.6 ± 13.1 years (mean ± SD) (Table 1). The most common cancer types were gastrointestinal (24%), genitourinary (12%), breast (11%), skin (8%), respiratory/thoracic (9%), musculoskeletal (6%), gynecologic (5%), and endocrine (2%).

**Table 1:** Cohort demographics.

	Total APG Cohort (*N* = 2141)	Non‐CFTR variant (*N* = 2070)	CFTR Variant (*N* = 71)	*p* value
Sex (female; number, %)	1032 (48.2)	999 (48.3)	33 (46.5)	0.8098
*Self identified race (number, %)*				0.9059
White (European)	1867 (87)	1802 (87)	65 (92)	
Black (AA)	156 (7.3)	153 (7.4)	3 (4.2)	
Asian	35 (1.6)	34 (1.6)	0 (0)	
American Indian, Alaskan or Hawaiian Native	5 (0.2)	5 (0.2)	1 (0.14)	
Multiple	4 (0.2)	4 (0.2)	0 (0)	
Unreported	74 (3.4)	72 (3.6)	2 (2.8)	
*Cancer type (number, %)*				0.0231
Gastrointestinal	515 (24)	491 (23.7)	24 (34)	
Genitourinary	254 (11.9)	245 (11.8)	9 (13)	
Breast	232 (11)	223 (11)	9 (13)	
Respiratory/thoracic	197 (9)	192 (9)	5 (7)	
Skin	75 (3)	69 (3)	6 (8.4)	
Endocrine and neuroendocrine	45 (2)	43 (2)	2 (2.8)	
Neurologic	70 (3)	67 (3)	3 (4.2)	
Musculoskeletal	124 (5.8)	120 (5.8)	4 (5.6)	
Head and neck	70 (3)	67 (3)	3 (4.2)	
Gynecologic	85 (4)	81 (4)	4 (5.6)	
Germ cell	2 (< 0.1)	2 (< 0.1)	0 (0)	
Miscellaneous or primary cancer type undetermined	472 (22)	470 (23)	2 (3)	
Age at cancer diagnosis (years) (mean ± SD)	57.6 ± 13.1	57.6 ± 13.1	57.7 ± 10.9	0.9493

**Figure 2:**
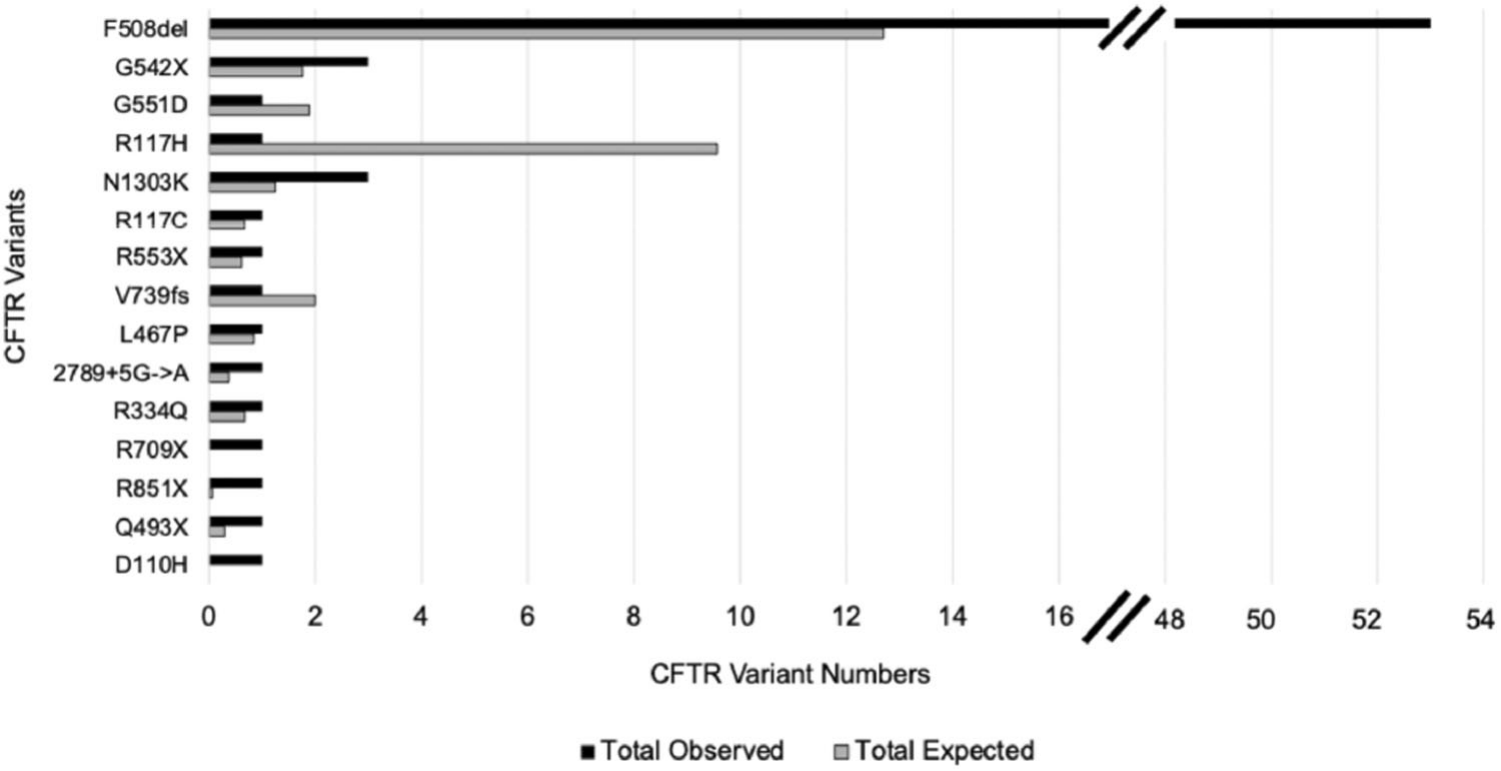
CFTR Pathogenic Variants (PVs) Predicted and Observed. The y‐axis displays all CFTR PVs present observed (APG cohort) shown with light gray bars and the expected (calculated based on Allele Frequency Aggregator (ALFA)) shown in the dark black. Each CFTR variant has both an expected and observed, however if one of these is zero, then it is not visible. The F508del PV was observed significantly more than expected and the R117H PV was observed significantly less than expected using a two‐sample proportion test.

Based on known MAFs and the racial distribution of our cohort, we would have expected 33 people (1.5%) to be carriers of a pathogenic *CFTR* variant. However, *CFTR* PVs were present in 71 people (3.3%) (*p* < 0.001). There was only one PwCF (homozygous F580del) with a clinical diagnosis of CF in the cohort. Nineteen unique pathogenic *CFTR* variants were identified in our cohort (Figure 2). The F508del was the most common variant observed, accounting for 75% of the PVs; the remaining 25% of the cohort included 14 unique variants. We observed a fourfold enrichment of F508del in our cohort based on 53 patients having the variant compared to an expected frequency of 12.7 subjects (*p* < 0.001).

The demographics of the total APG cohort and those with *CFTR* PVs were not significantly different with regard to sex, race, or age at diagnosis, but the distribution of cancer types was significantly different between the total APG cohort and those with *CFTR* PVs (Table 1). The observed frequencies of *CFTR* PVs were higher than expected for skin and gastrointestinal cancers (2.4‐fold and 1.4‐fold enrichment, respectively, with standardized residuals ≥ 2) and lower than expected for the miscellaneous or primary cancer type undetermined category (0.1‐fold enrichment and standardized residual of –4.0).

Of the 71 patients with *CFTR* PVs, two patients had PV in other genes with either an established or possible association with increased cancer risk consisting of the L2357 frameshift variant in *BRCA2* (rs80359636) and the Y179C missense variant in *MUTYH* (rs34612342). Sixty‐seven patients had 1+ somatic variant with established cancer pathogenicity, including variants in 142 genes; the median number of somatic cancer risk variants was 3 (range: 1–19). The prevalence of somatic cancer risk variants in *CFTR* PV carriers was compared to that from the COSMIC database [24]. When considering all cancer types, pathogenic somatic variants in *CDKN2A* (identified in 12%.7 of *CFTR* PV carriers; Sidak‐corrected *p* = 0.0069) and *CDKN2B* (identified in 4.2% of *CFTR* PV carriers; Sidak *p* = 0.0428) were more common in our cohort of *CFTR* PV carriers than in the global COSMIC database. When considering individual cancer types (and limiting to genes with pathogenic somatic variants seen in more than one *CFTR* PV carrier with that cancer type), the following genes more frequently had pathogenic somatic variants in *CFTR* PV carriers than in the corresponding cancer types in the COSMIC database: *KIT* in GI tract (identified in 66.7% of *CFTR* PV carriers with GI cancers; Sidak *p* = 0.0007); and *KRAS* in esophageal (40.0%; Sidak *p* = 0.0023). Pathogenic somatic enrichment data for all genes and in all cancer types are presented in Supporting Information S1: File 1; genes and associated cancer types with significant *p* values after Sidak correction are bolded, with p‐values highlighted in red.

Supporting Information S1: File 1. This table contains all pathogenic and somatic enrichment data for all genes and for all cancer types. Genes with significant p‐value after Sidak correction are bolded with p‐values highlighted in red.

## 4 Discussion

The prevalence of *CFTR* PVs in people with solid tumors was 2.2‐fold higher than expected based on the racial distribution of our cohort. In our cohort, F508del made up 75% of the PVs, but the other 25% of the cohort included 14 unique variants. In a previous study, Shi et al., identified over 14000 subjects included in the UK Biobank that had a heterozygous *CFTR* F508del PV, but did not search for any of the other nearly 500 PVs [12]. They found that the F508del occurred at a higher rate in subjects with a confirmed cancer diagnosis as compared to those without a cancer diagnosis, and that F508del rates were highest in subjects with colorectal cancer, but also significantly higher in cancers of gallbladder and biliary tract, thyroid cancer and unspecified non‐Hodgkin's lymphoma. These findings of a 1.4‐fold enrichment of *CFTR* PVs in patients with gastrointestinal cancers is consistent with cancer risk in people with CF as data indicate that colorectal cancer incidence is up to 10 times greater and occurring at a younger age in people with CF as compared to the general population [3]. These data in people with CF prompted the CF Foundation to commission a practice guideline for increased colorectal cancer screening for people with CF [4], yet, there are no recommendations of enhanced screening for people with only one CFTR PV.

The prevalence of skin cancers in our *CFTR* cohort was also higher compared to the overall AGP cohort suggesting that *CFTR* PVs may increase skin cancer risk. Conversely, in a case control study of 574 lung cancer patients and 679 control patients, *CFTR* F508del was associated with a 68% reduced risk for lung cancer [13]. Our data are consistent with this as 9% of our APG cohort had a respiratory or thoracic cancer diagnosis and this cancer type only represented 7% of the cancers in the *CFTR* PV cohort.

CFTR may drive cancer via several different mechanisms including regulation of ion transport across cellular membranes, an increase in pro‐inflammatory cytokines and chemokines which can promote a pro‐tumorigenic environment, and involvement in proliferation and cell survival pathways [11]. CFTR has been shown to be involved in cellular proliferation pathways including epithelial–mesenchymal transition (EMT), which is a latent developmental process, that can be re‐activated in fibrosis and cancer [25]. In cells and tissues expressing PVs, EMT was active shown by destructured epithelial proteins, defective cell junctions, increased levels of mesenchymal markers and EMT‐associated transcription factors, and hyper‐proliferation and impaired wound healing [25]. Data suggests that CFTR may play a role in tumor resistance to chemotherapy [26]. This may be significant as our cohort included only patients that had whole genome sequencing as part of their clinical cancer care which tend to be patients that have failed first line chemotherapy or present with an advanced cancer.

Interestingly, people with PV CFTR demonstrated a lower prevalence of respiratory system cancer patients with CFTR PV (7% vs. 9% in those without PV CFTR variants). Due to the small sample size it is unclear whether this could signal a potential protective role of CFTR PV against lung cancer, but should be further evaluated in future studies.

Our study is limited by the lack of a control “non‐cancer” cohort with available genetic sequencing data. While this limits the generalization of these findings, this initial report is important to justify future studies. To further research cancer risk associated with *CFTR*, germline sequencing data from a cohort that includes both people with known cancer diagnosis and healthy controls is necessary. To investigate the interplay of *CFTR* and other known cancer risk variants, it is necessary to use a large diverse genetic database such as All of Us or UK Biobank [27, 28]. UK Biobank consists of subjects that are on average healthier than the average UK citizen [28]. Though the UK Biobank contains mostly people of European ancestry, it contains ~8000 subjects of African ancestry in addition to other ancestry backgrounds, enabling our work to apply to diverse populations. All of Us is a more diverse database in age, ethnicity, and disease compared to our APG cohort and UK Biobank [27]. In addition to *CFTR* variants, our cancer cohort had a high frequency of other known somatic cancer risk variants which could have been the major driver of cancer, or alternatively, *CFTR* may have added an increased risk variant [29].

Additionally, because of the nature of our cancer precision genomics program, clinical genomic sequencing is not always performed in early stage cancers, rather performed with higher frequency when patients fail initial cancer treatments. For this reason, our cohort is enriched for patients with progressive and treatment resistant cancers which could suggest that *CFTR* dysfunction may increase treatment resistance and is enriched in late‐stage patients. Additionally, the classification of *CFTR* variants and their pathogenicity are not well defined and can conflict between databases.

Lastly, the numbers of some of the cancer types were low and dominance of the F508del may have overshadowed other PV therefore results may not be reflective of a larger cohort and warrant further investigation.

The results we have presented here support further investigation of *CFTR* and cancer risk in a larger diverse cohort and will help to inform methods for future analysis. Understanding of *CFTR* dysfunctions' role in cancer risk is important to advise people with CF on family planning and potential need for enhanced cancer prevention strategies, but also as *CFTR* modulator therapies become more affordable, these therapies may have a role in cancer prevention in people with only one *CFTR* variant.

## Author Contributions

**Tyler Shugg:** conceptualization, investigation, writing − original draft, methodology, writing − review and editing, formal analysis. **Katherine A Gallaway:** investigation, writing − original draft, writing − review and editing. **Nick Powell:** conceptualization, formal analysis, writing − review and editing. **Todd C. Skaar:** conceptualization, writing − review and editing. **Steven M. Bray:** conceptualization, validation, methodology, formal analysis. **Leigh Anne Stout:** data curation, writing − review and editing, methodology. **Abi Colwell:** Investigation, writing − review and editing. **Cynthia D. Brown:** conceptualization, writing − review and editing. **James E. Slaven:** formal analysis, writing − review and editing. **Emma M. Tillman:** conceptualization, investigation, methodology, data curation, supervision, resources, writing − original draft, funding acquisition, writing − review and editing, formal analysis, project administration.

## Conflicts of Interest

The authors declare no conflicts of interest.

## Supporting information

Supplemental File 1 2.

## Data Availability

The data that supports the findings of this study are available in the Supporting Information Material of this article.
